# The critical roles of bioactive sphingolipids in inflammation

**DOI:** 10.1016/j.jbc.2025.110475

**Published:** 2025-07-11

**Authors:** Ana Gomez-Larrauri, Asier Larrea-Sebal, César Martín, Antonio Gomez-Muñoz

**Affiliations:** 1 Department of Biochemistry and Molecular Biology. Faculty of Science and Technology. University of the Basque Country (UPV/EHU), Bilbao, Spain; 2 Respiratory Department. Cruces University Hospital, Barakaldo, Bizkaia, Spain; 3 Biofisika Institute (UPV/EHU, CSIC), Leioa, Bizkaia, Spain

**Keywords:** apoptosis, cell growth, cancer, ceramide, ceramide kinase, ceramide 1-phosphate, inflammation, sphingosine, sphingosine kinase, sphingosine 1-phosphate, sphingolipids

## Abstract

The bioactivity of sphingosine (Sph), ceramides, sphingosine 1-phosphate (S1P) and ceramide 1-phosphate (C1P) has been known for decades. However, the molecular mechanisms by which these sphingolipids exert their biological actions are not completely understood. Initial studies showed that Sph inhibited protein kinase C and phosphatidate phosphohydrolase activities paving the way for further discoveries on the key role these sphingolipids play in signal transduction processes. Soon after the implication of Sph in cell signaling events, it was shown that ceramides were also able to regulate relevant cell functions, including cell death and survival, differentiation, autophagy, and inflammation. Subsequent studies showed that both Sph and ceramides could be phosphorylated in cells and that S1P and C1P counteracted many of the actions elicited by ceramides. Both phosphorylated sphingolipids are essential for regulation of many physiological and pathological cell processes. The present review has been undertaken to highlight and clarify the molecular mechanisms and signaling pathways that are regulated by Sph, ceramides, S1P and C1P in cells with special attention been paid to understand the role of these bioactive sphingolipids in inflammatory responses and inflammation-associated diseases.

Sphingolipids have long been known to be essential components of cell membrane architecture, with the structural polar phospholipid sphingomyelin (SM) being the most abundant in mammalian cell membranes and the myelin sheath surrounding the nerves. In cell membranes, SM contributes to 10 to 20% of total lipids, and is particularly concentrated in the outer leaflet of lipid rafts (reviewed in (1)), which are thought to be relevant membrane domains for cell signaling and protein trafficking events (2). In addition to harboring cholesterol, lipid rafts also contain relatively high concentrations of sphingolipids namely glucosphingolipids such as cerebrosides, a class of sphingolipids bearing a sugar moiety, and gangliosides, which bear oligosaccharide chains and sialic acid in their molecules, and which can also regulate cell signaling processes (2). Other important functions of gangliosides are participation in host recognition by the immune cells (3), modulation of the functions of growth factors, interferon, or insulin by binding to either the agonist or to its receptor (4), and facilitation of bacterial toxin entry into cells, such as for instance incorporation of cholera toxin (5). Apart from SM, cerebrosides, and gangliosides, which are all complex sphingolipids, the simple sphingolipids Sph, ceramide and their phosphorylated forms, sphingosine 1-phosphate (S1P) and ceramide 1-phosphate (C1P) are known to be key regulators of crucial biological cell functions.

Sph is an essential component of sphingolipids and sphingolipid metabolism, serving as direct precursor of two major bioactive sphingolipids, ceramides, and S1P, which are mainly synthesized by ceramide synthases (CerS) or sphingosine kinases (SphKs), respectively (Fig. 1). Sph becomes concentrated at specific locations within the plasma membrane by the action of flotillin oligomers, which also induce recruitment of SphK2 at the plasma membrane and late endosomes to facilitate S1P generation (6). Sph can exert important cellular signaling activities, being able to modulate the activities of relevant signal transduction enzymes, including protein kinase C (PKC) (7); phosphatidate phosphohydrolase-1 (PAP-1, known as lipin) (8); diacylglycerol kinase (DAGK) (9); or phospholipase D (PLD) (10), which are involved in the regulation of lipid metabolism and cell growth and survival. In particular, Sph promotes apoptosis by modulating the phosphorylation of some transcription factors, causing an imbalance in the levels of Bcl-2 family of proteins (11, 12). In addition, Sph possesses bactericidal or bacteriostatic properties, being also able to kill a number of different types of pathogens, including viruses and fungi (13).

**Figure 1:**
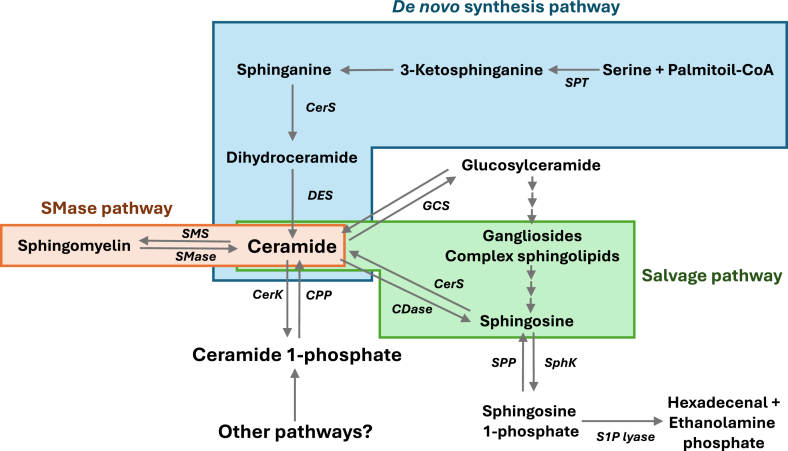
**Metabolic pathways of bioactive sphingolipids.** Ceramides are the central core of sphingolipid metabolism. They can be synthesized by three major pathways: (1) the *de novo* synthesis pathway, in which serine palmitoyl-CoA transferase (SPT) and ceramide synthase (CerS) are the major regulatory enzymes of the pathway; (2) the sphingomyelinase (SMase) pathway, in which ceramides are generated directly from degradation of sphingomyelin (SM) by different SMases. The reversed reaction is catalyzed by SM synthase (SMS); (3) the salvage pathway, where sphingosine that is derived from the metabolism of complex sphingolipids is recycled back to ceramide by the action of CerS activity. Once generated, ceramide can be phosphorylated by ceramide kinase (CerK) to yield C1P, or it can be degraded by ceramidases (CDases) to form Sph. Subsequently S1P is synthesized through phosphorylation of Sph by sphingosine kinases (SphK). The reversed reaction is catalyzed by S1P phosphatases (SPP), or LPPs. S1P lyase degrades S1P to 2-trans hexadecenal and ethanolamine phosphate. C1P, ceramide 1-phosphate; LPP, lipid phosphate phosphatase; S1P, sphingosine 1-phosphate; SM, sphingomyelin.

Ceramide is the core of complex sphingolipids. In addition to its involvement in cell membrane architecture, it is a second messenger involved in the regulation of many biological functions including cell growth and survival, autophagy, cell differentiation, and inflammatory responses (14, 15, 16).

Regarding C1P and S1P both metabolites exert different, and often opposing, effects to their nonphosphorylated counterparts, suggesting that the kinases responsible for their biosynthesis may be highly regulated. Although Sph and ceramides signal for cell cycle arrest and apoptosis, C1P and S1P are potent stimulators of cell growth and inhibit apoptosis (17, 18, 19). Noteworthy, both C1P and S1P are heavily implicated in inflammatory responses, although the role of these two phosphosphingolipids in inflammation is controversial. In particular, while C1P promotes inflammation in various cell types, it blocks infiltration of immune cells in the lungs *in vivo*, it inhibits the secretion of proinflammatory cytokines from lung cells, and it reduces emphysema in mice exposed to cigarette smoke (reviewed in (20)). Likewise, S1P can trigger immune responses leading to neuroinflammation (21), or it can exert anti-inflammatory actions by promoting anti-inflammatory cytokine release and inhibition of proinflammatory mediators (20).

The regulatory properties and mechanisms of action of the simple sphingolipids are discussed below.

## Sphingosine

Sph, the simplest sphingolipid of all, is exclusively produced by catabolism of complex sphingolipids (22, 23, 24, 25, 26); no anabolic pathway has so far been associated with Sph biosynthesis in mammalian cells (Fig. 1). Sph became important in cell biology with the discovery that it regulated the activities of PKC (7), PAP (8), PLD (10), and DAGK (9), which are all key regulatory enzymes of lipid metabolism and cell signaling processes, and are implicated in many biological functions including proliferation, survival, differentiation, or cell migration (27, 28, 29). After these important discoveries, Sph was implicated in the regulation of various other cellular processes, both physiologically and pathologically, including cell growth and death, autophagy, and development (30, 31). In addition, Sph plays roles in the regulation of actin cytoskeleton and endocytosis, and more recently it has been described as antimicrobial for both Gram-positive and Gram-negative bacteria (32), enveloped viruses (33), and fungi (34). The antimicrobial actions of Sph have been described in various different tissues such as the respiratory epithelium, the skin, or the oral cavity (reviewed in (13)). Of interest, inhalation of nebulized Sph has been shown to be effective at preventing or ameliorating pneumonia in multiple cystic fibrosis patients and mouse models (35, 36, 37). Noteworthy, the levels of Sph are reduced in lung epithelial cells of mice with cystic fibrosis, a fact that has been associated with high infection susceptibility (37, 38, 39). In particular, Sph has been shown to have antibacterial activity against *Staphylococcus aureus*, and opportunistic Gram-positive bacterium that causes severe respiratory tract and systemic infections, and *Pseudomonas aeruginosa*, and opportunistic Gram-negative bacterium commonly associated with pneumonia and other pulmonary diseases such as chronic obstructive pulmonary disease (COPD), cystic fibrosis, or sepsis (40), although the latter actions occurred at pharmacological concentrations of the sphingolipid. Sph is also effective at killing *Neisseria gonorrhoeae*, a Gram-negative pathogen that causes gonorrhea, a sexually transmitted disease that has the potential to cause severe infections associated with endocarditis and arthritis (41, 42, 43). Under a mechanistic point of view, Sph incorporates into the membranes of *N. gonorrhoeae*, leading to decreased survival of bacteria.

In addition to these remarkable antibacterial effects, Sph has also been shown to be effective at preventing virus and fungi infections. The first report showing that targeting sphingolipid metabolism could prevent virus replication was published by Sakamoto et al., who found that a lipophilic long-chain base compound (NA255) that inhibited *de novo* synthesis of sphingolipids could directly interact with SM to prevent infection by hepatitis C virus (33). Since then, many other viruses, including human immunodeficiency virus (HIV), Ebola virus, measles virus, and rhinovirus have been shown to enter the cells through a mechanism involving sphingomyelinase (SMase) activation. Upregulation of SMases produces ceramides, which the viruses use to adhere and penetrate the host cell (44, 45, 46, 47). More recently, Sph has been demonstrated to block propagation of herpes simplex virus in macrophages (48), and to protect against infection of the severe acute respiratory syndrome coronavirus-2 (SARS-CoV-2) that causes coronavirus disease 2019 (COVID-19). Specifically, Sph blocked the interaction of the viral spike protein with ACE2 receptors, which the SARS-CoV-2 virus uses for infecting the host cells (49). Concerning its antifungal properties, Sph was shown to be effective against infections by *Candida albicans* (50), a polymorphic fungus that can cause infections ranging from superficial alterations of the skin to life-threatening systemic infections (51).

## Sphingosine 1-phosphate

**Figure 2:**
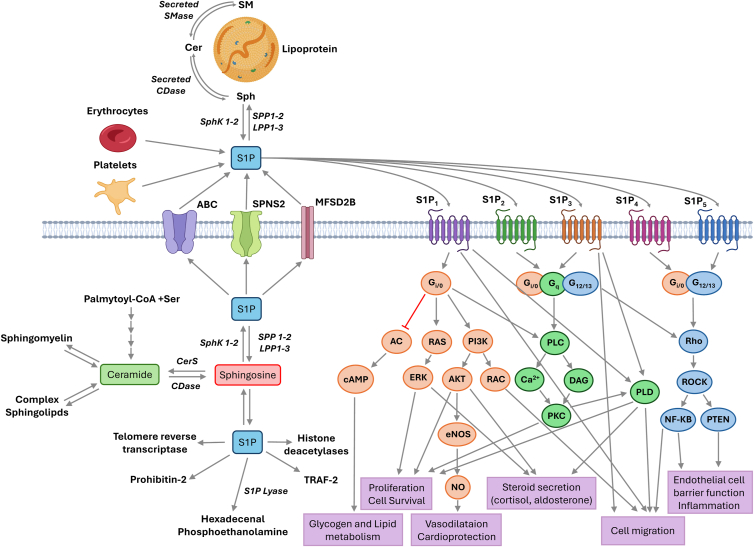
**Biological actions of S1P in mammalian cells.** S1P can be synthesized intracellularly by the action of sphingosine kinases (SphKs) leading to stimulation of tumor necrosis factor-α-receptor-associated factor 2 (TRAF-2), telomere reverse transcriptase, prohibitin-2 or histone deacetylase activities. Intracellular S1P can be transported to the extracellular milieu by the ATP-binding cassette (ABC) family of transporters, sphingolipid transporter-2 (SPNS2), or major facilitator superfamily domain containing 2B (MFSD2B) transporter. The extracellular S1P pool can be increased by the action of extracellular SphKs acting on lipoprotein-derived Sph and by secretion of S1P from erythrocytes and platelets. Extracellular S1P can then interact with a family of five different G protein–coupled receptors (S1PR1-5) to regulate a variety of cell functions including glycogen and lipid metabolism, cell proliferation and survival, steroid hormone (cortisol and aldosterone) secretion, cell migration, vasodilatation, or participation in inflammatory responses. S1P, sphingosine 1-phosphate.

Another important role of Sph in cell biology is serving as substrate for SphK1 and SphK2, to produce S1P (Fig. 1). SphK1 is a cytosolic enzyme whereas SphK2 is mainly localized to the nucleus. The differential localization of the SphKs allows these enzymes to regulate distinct but crucial cell functions. For example, in the cytoplasm, the axis SphK1/S1P regulates autophagy and protein ubiquitination in neurons, while nuclear SphK2/S1P regulates gene transcription (52). S1P also regulates other relevant cellular processes, including proliferation, survival, differentiation, or cell migration (53, 54, 55, 56), in both physiological and pathological settings. S1P is found in the plasma and lymph. It is produced and secreted in abundance by erythrocytes and platelets and its levels are supplemented by endothelial cells (57, 58). Transport of intracellular S1P to the extracellular milieu is regulated by selective transporters of the ATP-binding cassette (ABC) family, sphingolipid transporter 2 (SPNS2), or major facilitator superfamily domain containing 2B (MFSD2B) (Fig. 2) (59). However, S1P can also be transported from the extracellular compartment back into cells by the cystic fibrosis transmembrane conductance receptor (60). Although the half-life of S1P is very short (ranging from 1 to 15 min) (61, 62) and so, it is rapidly cleared from the circulation by degradative enzymes, S1P can be quickly synthesized to maintain adequate plasma concentrations (63). The major S1P catabolizing enzymes are the pyridoxal 5-phosphate-dependent S1P lyase, which irreversibly degrades S1P to hexadecenal and ethanolamine phosphate, S1P phosphatases 1 and 2, and the lipid phosphate phosphatase 3 (LPP3) (Fig. 1) (64), which together with LPP1 and LPP2 were initially designated phosphatidate phosphohydrolase-2 (PAP-2) and that participate in relevant cell signaling processes (18, 28, 29, 65). Noteworthy, an important physiological action of S1P, which has often been overlooked, is the regulation of cortisol and aldosterone secretion, which are hormones that regulate vital metabolic processes (66, 67, 68). The mechanisms by which S1P stimulates steroid hormone secretion involve prior upregulation of PLD, PAP, and PKC activities, as well as activation of the mitogen-activated protein kinases extracellularly regulated kinase (ERK) 1 and 2 and phosphatidylinositol 3-kinase (PI3K) (66, 67, 68).

Contrary to Sph, many of the biological actions that are elicited by S1P take place through interaction of S1P with five different cell membrane receptors, which are designated S1PR1-5 (Fig. 2). The five S1P receptors belong to the G protein–coupled receptor (GPCR) family and are coupled to different heterotrimeric G proteins, namely Gs, Gi/o, Gq/11, or G12/13, and can be activated by S1P in a paracrine or autocrine manner (69). Nevertheless, besides receptor stimulation, S1P can also exert intracellular (receptor-independent) actions. For example, S1P can bind and regulate the activities of tumor necrosis factor-α-receptor-associated factor 2 (TRAF-2), a cytosolic signal transduction mediator protein (70), atypical protein kinase C (71), telomere reverse transcriptase (72), prohibitin-2, a protein that affects mitochondrial respiration (73), or histone deacetylases, which regulate gene expression in the nucleus (74).

Apart from regulating physiologic cell functions, S1P has been associated with pathology, including neurological disorders such as stroke and aneurysmal subarachnoid hemorrhage (75), and it participates in inflammatory responses and lymphocyte extravasation to sites of inflammation, namely through binding to S1PR1 (76). Concerning inflammation, S1P has been associated with the migration of neural progenitor cells and to modulate inflammation in the brain (77). Also, S1P is elevated in psoriasis patients (78), and plays a major role in regulating airway inflammation and bronchoconstriction in asthmatic individuals. In fact, relatively high concentrations of S1P in the airways cause bronchoconstriction and provoke airway hyperresponsiveness, leading to exacerbation of asthma (79). Furthermore, S1P induces the migration of immune cells into the lungs causing or enhancing inflammation, thereby contributing to the development of respiratory diseases (79). A relevant role of S1P in inflammatory bowel diseases, namely in ulcerative colitis, is also well established (80). In fact, two drugs that resemble S1P structure and antagonize S1P receptors, ozanimod and etrasimod have been approved for the treatment of ulcerative colitis (81, 82). In particular, ozanimod is highly selective for binding to S1PR1 and 5 receptors (83), whereas etrasimod shows high affinity for binding to S1PR1, 4, and 5 (84). In this connection, it should be emphasized that fingolimod (FTY720), a Sph analog that becomes phosphorylated when incorporated into cells, acts as S1PR1 antagonist to inhibit lymphocyte migration and inflammatory responses in the brain (85). Noteworthy, FTY720, which ameliorates brain injury through multiple mechanisms and is a strong candidate for stroke treatment (85), was the first Food and Drug Administration-approved nonselective S1P modulator used for the treatment of relapsing-remitting multiple sclerosis (86, 87). S1P also promotes inflammation through activation of the S1PR4, which is highly expressed in hematopoietic cells. Activation of S1PR4 induces neutrophil migration to sites of inflammation, regulates antigen presentation by dendritic cells and promotes inflammation by polarization of T-helper 17 cells (88). However, participation of S1PR4 in inflammation is controversial as activation of this same receptor has been associated with anti-inflammatory effects of S1P. Specifically, it was shown that upregulation of S1PR4 caused secretion of the anti-inflammatory cytokine interleukin (IL)-10 (89), and to reduce secretion of proinflammatory interferon-γ (90). Also, the well-established anti-inflammatory properties of high-density lipoproteins (HDLs) have been associated with the high levels of S1P that are bound to the apoM component of the lipoprotein particle (91). Up to 80% of the plasma S1P is bound to the HDL particles, with the remaining portion of S1P being associated with albumin. Like S1P, HDL was shown to induce phosphorylation (activation) of signal transducer and activator of transcription-3 (STAT3), which is a mediator of tumor-promoting inflammation and increases tumor cell proliferation (92), and to inhibit executive proapoptotic caspase 3 and surviving expression, which are key regulatory proteins of apoptosis. Interestingly, the latter effects were mimicked by apoM but not by HDL that was deprived of S1P (93). The controversial participation of S1P in inflammation is also evident when looking into the role of S1P in controlling vascular permeability in endothelial cells, a condition that is strongly associated with formation of edema and inflammation. Increased permeability is an important feature of airway inflammation, including asthma and COPD, and is also associated with inflammatory bowel disease, arthritis, ischemic stroke, infections, cancer, and other conditions where leakage from blood vessels into the interstitial space can result in fluid accumulation or edema (94). The effect of S1P on increasing vascular permeability in endothelial cells is mediated by interaction with its S1PR2, as demonstrated by Sanchez *et al.* (95). However, the same group reported that activation of S1PR1, led to inhibition of vascular leakage and edema (95). Mechanistically, in endothelial cells, S1P binding to S1PR1 and S1PR3 led to stimulation of PI3K/Akt and subsequent activation of the homomeric G protein Rac whereas binding of S1P to S1PR2 activated phosphatase and tensin homologue (PTEN), a phosphatase that antagonizes PI3K activity, leading to inhibition of Rac. The opposite effects of S1PR1 and S1PR2 can be explained by the antagonistic activities of their downstream effectors, PI3K and PTEN, respectively (95). From these data, it was proposed that changes in the balance of S1PR1 and S1PR2 in the endothelium may determine the regulation of vascular permeability by S1P (95). For details on the regulatory actions of S1P the reader is referred to elegant reviews by S Spiegel, T Hla, J Saba, and M Nawajes (79, 96, 97, 98). In addition to this, a number of reports by Saba´s group highlight the importance of S1P lyase in the biology of S1P, showing that this enzyme is crucial for regulating many of the S1P biological actions including immune cell trafficking, angiogenesis, or cell transformation (Reviewed in (99)). S1P lyase is a pyridoxal 5′-phosphate dependent enzyme that is located in the outer leaflet of the endoplasmic reticulum (ER) and irreversibly cleaves S1P into the long-chain aldehyde hexadecenal and ethanolamine phosphate in the final step of sphingolipid catabolism (99, 100). The importance of S1P lyase is also highlighted in the sphingosine phosphate lyase insufficiency syndrome, a genetic disorder associated with neurological, endocrine, renal, skin, and immune system alterations that is caused by mutations or dysfunction of S1P lyase (79).

Another major metabolite of Sph, which can be generated by metabolism of complex sphingolipids in the salvage pathway, is ceramide. The latter can also be synthesized by the *de novo* pathway, which takes place in the ER, and by direct degradation of SM in the plasma membrane by acidic or neutral SMases (ASMase or NSMase, respectively) (Fig. 1), or by degradation of SM by secretory SMases that are present in low-density lipoprotein (LDL) particles (101). Ceramides have also been shown to occur in the liver mitochondria through reverse ceramidase activity (102) although this pathway has not been exhaustively explored. Moreover, ceramides can be generated through dephosphorylation of C1P by the action of selective phosphatases, including specific C1P-phosphatase (103) or promiscuous LPPs (29), which can also dephosphorylate phosphatidic acid (PA), lysophosphatidic acid, and S1P (104), or by β-glucosylceramidases acting on glucosylceramide to remove the sugar moiety of the cerebroside to release ceramide (101).

## Ceramides

Ceramides are particularly important because they are central in sphingolipid metabolism and are the core moiety of complex sphingolipids. Structurally, ceramides are formed by condensation of a 16-carbon chain fatty acid (palmitic acid) and the 3-carbon amino acid serine, followed by decarboxylation and incorporation of a (usually) long-chain fatty acid that is attached to the 18-carbon chain moiety through an amide bond to generate dihydroceramide. Then, a desaturase introduces a double bond in position 4 to 5 *trans* to form ceramide, in the *de novo* synthesis pathway (Fig. 1). Two major regulatory enzymes of this metabolic route are serine palmitoyl transferase (SPT) which catalyzes the first and rate-limiting step of the pathway, and CerS, which catalyze the incorporation of fatty acids of distinct hydrocarbon chain lengths into the molecule to form dihydroceramides (Fig. 1). Many of the studies to delineate the *de novo* ceramide synthesis were carried out using two selective inhibitors, myriocin, which was isolated from culture broths of the fungus *Isaria sinclairii*, and fumonisin B1, a toxin produced mainly by the fungi *Fusarium proliferatum* and *Fusarium verticillioides*, and which potently block the activities of SPT (105, 106) and CerS (107, 108, 109), respectively. However, care should be taken when using these inhibitors so as to avoid nonspecific effects. For example, relatively high concentrations of myriocin can stimulate perilipin protein 2, an enzyme that increases lipid droplet formation and is implicated in the clearance of *Mycobacterium tuberculosis* through a mechanism independent of ceramide reduction (110), and might also induce other off-target effects at high concentrations. Also, fumonisin B1 can induce off-target effects, such as production of reactive oxygen species leading to DNA damage, upregulation of proinflammatory tumor necrosis factor alpha (TNFα) and IL-1β expression, and induction of toxic effects leading to carcinogenesis through mechanisms that are independent of ceramide inhibition (26, 108, 111).

In addition to their important role in cell membrane architecture, ceramides are key regulators of vital cellular functions. However, dihydroceramides, which only differ from ceramides by lacking the 4 to 5 trans double bond in the Sph backbone of the molecule (22), were reported to be inert. Nevertheless, the latter hypothesis was challenged after the discovery that contrary to the initial studies using short-chain dihydroceramides, the natural long-chain dihydroceramides were also bioactive, being implicated in the regulation of autophagy, apoptosis, oxidative stress, cancer, or metabolic diseases (112, 113, 114). For details on the cellular actions of dihydroceramides, the reader is referred to a recent review by Y. Jang (115).

**Figure 3:**
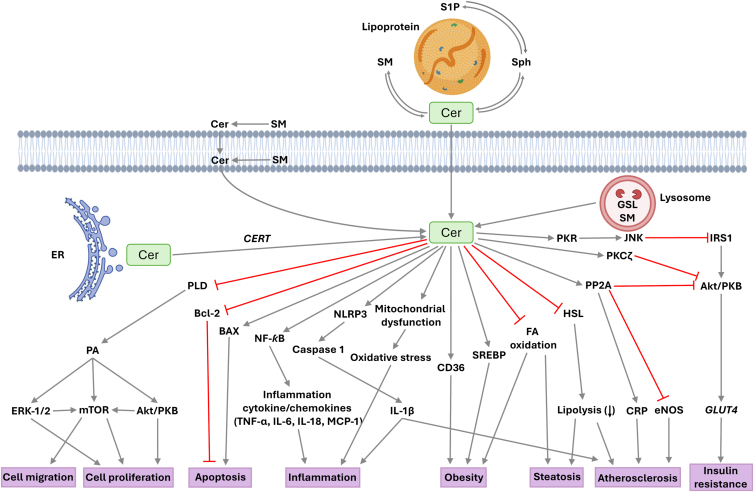
**Biological actions of ceramides in mammalian cells.** Accumulation of intracellular ceramides leads to inhibition of cell growth and stimulation of apoptosis through a variety of mechanisms including blockade of PLD, or inhibition of antiapoptotic Bcl-2 and activation of proapoptotic Bax proteins, respectively. Concerning inflammation, ceramides upregulate the NLRP3 inflammasome/caspase 1 axis, and cause mitochondrial dysfunction leading to proinflammatory cytokine production, being also implicated in the development of inflammation-associated diseases, such as obesity, steatosis, or atherosclerosis. Ceramides are also involved in insulin resistance through a mechanism involving Akt/PKB (protein kinase B) inhibition. PLD, phospholipase D.

Concerning ceramides, they have been demonstrated to induce cell cycle arrest and apoptosis (101, 116, 117), and to promote inflammation (Fig. 3) in a great variety of cell types. Although inhibition of cell division and induction of apoptosis can be beneficial actions, namely in contexts of hyperplasia or tumorigenesis, promoting inflammation may end up being detrimental for cells. In principle, inflammatory responses are beneficial for the organism, but they can be harmful when out of control.

In the context of inflammation, ceramides can bind to toll-like receptor (TLR)-4 to trigger inflammatory pathways, and can promote nucleotide-binding domain, leucine-rich-containing family, pyrin domain containing 3 (NLRP3) inflammasome activation (Fig. 3). The latter actions lead to the production of the proinflammatory cytokines IL-1β, IL-6, IL-18, and TNF-α in macrophages, both in a receptor-dependent or independent manner (118, 119). In particular, ceramides have been recognized to be key players in the pathogenesis of various inflammatory lung diseases, including asthma, COPD, pulmonary fibrosis (120, 121, 122, 123), and pulmonary infections (124). Although ceramides are primarily synthesized intracellularly, they have also been identified in biological fluids such as plasma and bronchoalveolar lavage fluid, where they associate with carrier proteins or lipid microvesicles derived from the plasma membrane (122). Notably, exogenous ceramides can induce ASMase activity and are able to stimulate the *de novo* synthesis pathway, suggesting the existence of a ceramide paracrine amplification loop that enhances intracellular ceramide levels (122). The role of ASMase-derived ceramides in pulmonary inflammation is highlighted by their involvement in platelet-activating factor (PAF)-induced pulmonary edema (125), as well as in the pathology of cystic fibrosis (123). The latter disease is characterized by chronic pulmonary inflammation, impaired mucociliary clearance, and increased susceptibility to infection. Also, accumulated ceramides in cystic fibrosis lungs have been linked to increased cell death, increased susceptibility to infections, and exacerbation of inflammation (126). Furthermore, exposure to cigarette smoke—a condition widely acknowledged as the primary cause of emphysema—elevates ceramide levels in human and murine lungs, as well as in primary endothelial and epithelial cells, and alveolar macrophages (127, 128, 129, 130). Increased ceramide concentrations have also been associated with alveolar endothelial and epithelial cell apoptosis, macrophage activation, and matrix proteolysis, all of which are hallmarks of emphysema. In line with the latter observations, NSMase has been implicated in cigarette smoke-induced epithelial cell apoptosis leading to emphysema (128, 129). Regarding infection, seminal studies by Grassmé *et al.* first established the role of ceramides and ASMase in bacterial infection. Their research work on *N. gonorrhoeae*, a pathogen primarily responsible for sexually transmitted infections, provided initial insights into this mechanism (131). Further investigations demonstrated that ASMase-deficient macrophages exhibited an impaired ability to resolve infections by *Salmonella typhimurium* (132), and ASMase KO mice displayed heightened susceptibility to *S. typhimurium* infection (133). Similarly, ASMase (−/−) mice were more prone to pulmonary infection by *P. aeruginosa* (124) but exhibited increased resistance to lethal *Mycobacterium avium* infections compared to WT mice (133). Nevertheless, it should be taken into account that ASMase deficient animals may enhance sphingolipid storage diseases, namely A or B Niemann-Pick disease (118, 119) adding difficulty to understand the regulatory functions of ASMase. Concerning asthma, epidemiological studies have identified increased adiposity as a risk factor for development of the disease. Obesity induces significant alterations in the mechanical properties of the respiratory system, potentially contributing to the onset of asthma (134), where eosinophils often play critical roles in exacerbation of the disease (135). However, obesity-related asthma does not correlate with eosinophilic airway inflammation, and low levels of the adipokine adiponectin have been associated with asthma in pediatric populations (136). In addition, leptin—another major adipokine—has been shown to stimulate the production of proinflammatory cytokines such as TNF-α, IL-6, and interferon gamma (IFN-γ), all of which are implicated in the pathology of asthma (137, 138). Although the precise mechanisms linking obesity and asthma remain unclear, systemic inflammation in obesity may potentiate asthmatic signaling cascades, with adipokines acting as modulators of these pathways (134, 139, 140).

Mechanistically, ceramides contribute to intracellular signaling by activating specific serine/threonine protein kinases (141, 142) or by stimulating serine/threonine protein phosphatases (PPs), including PP1, PP2A, and PP2C (143, 144, 145). In particular, activation of PP2A results in dephosphorylation and inactivation of Akt (protein kinase B, PKB) (146, 147), a downstream effector of PI3K, which pathway is crucial for promoting cell survival. This mechanism may partially explain the proapoptotic properties of ceramides (148). The PI3K/Akt signaling axis also plays a major role in insulin-mediated metabolic processes, and Akt inhibition contributes to insulin resistance and the pathogenesis of type II diabetes mellitus (149, 150). Type II diabetes is closely associated with obesity and increased adiposity, and is characterized by elevated ceramide concentrations in human plasma and mediastinal adipose tissue (151). In fact, there is compelling evidence supporting the role of ceramides as key mediators in obesity-associated type II diabetes (152, 153). In this context, ceramides were shown to facilitate the uptake and esterification of fatty acids, an action that was mediated by translocases such as CD36 (154, 155), and to induce the expression of sterol regulatory element–binding proteins (SREBP) (Fig. 3), a transcription factor that promotes the incorporation of free fatty acid into triglycerides to facilitate their storage in lipid droplets (156, 157). Moreover, ceramides can inhibit lipolysis by blocking the activation of hormone-sensitive lipase (Fig. 3) thereby also contributing to the development of obesity (154).

Circulating ceramides are predominantly transported in very low-density lipoprotein and LDL particles (158). Notably, LDL-associated ceramides have been implicated in promoting inflammation and skeletal muscle insulin resistance (118). High levels of both circulating and intracellular ceramides—particularly those generated in response to proinflammatory cytokines—can directly impair insulin-stimulated glucose uptake (118, 151, 158, 159). Plasma ceramide concentrations have been reported to approximate 11.5 μM (160). The interconnection between diabetes and inflammation is well established [65–72], and it is now evident that ceramides serve as a molecular link between obesity, diabetes, and inflammatory processes. Furthermore, recent studies have demonstrated that reductions in plasma ceramide levels—specifically C14:0-ceramides—correlate with exercise-induced improvements in insulin sensitivity in adult individuals (161). Also, under a mechanistic point of view, ceramides have been shown to induce the expression or activation of proinflammatory nuclear factor kappa B (NF-κB) (Fig. 3), a transcription factor that is ubiquitously expressed in mammalian cells (162). This transcription factor can regulate the expression of many genes involved in inflammatory responses such as, for instance, various cytokines or chemokines, including IL-1β, IL-6, IL-18, or monocyte/macrophage chemoattractant protein-1 (MCP-1), as well as genes encoding proinflammatory enzymes such as cyclooxygenase-2 (COX-2), which is involved in the production of proinflammatory eicosanoids, namely prostaglandins (162, 163). Alternatively, some inflammatory factors, including TNF-α, IL-1β, IFN-γ, or PAF can promote ceramide synthesis through activation of SMases thereby contributing to or exacerbating inflammation (19). Ceramides have also been shown to enhance lipopolysaccharide (LPS)-induced proinflammatory signaling through stabilization of TLR-4 (164) and specifically palmitoyl ceramide is elevated in systemic lupus erythematosus, a chronic autoimmune disorder that causes inflammation in the joints, skin, kidneys, heart, lung, brain and circulating blood cells (165). In skin, ceramides play a key role in the regulation of keratinocyte proliferation and differentiation, and constitute a hydrophilic extracellular lipid matrix that is indispensable for permeability barrier function (166). Although ceramides are vital for maintaining proper functionality of the skin, excessive ceramide accumulation may lead to inflammation (167). Specifically, and despite a decrease in ultra-long chain ceramides levels, very long chain ceramides are increased in psoriasis patients (168) and patients with psoriatic arthritis, an inflammatory arthropathy associated with psoriasis (169). In this connection, it has been recently reported that mechanistically, persistent inflammation mediated by saturated very long chain ceramides is largely dependent on sustained activity of REL (an NF-κB family transcription factor) (170), which is in agreement with other work showing the implication of NF-κB in the regulation of inflammatory responses by NSMase2 (171). Nonetheless, NF-κB-independent effects of ceramides have also been reported (172). Concerning inflammation, deletion of CerS2, the enzyme responsible for very long chain ceramide production limited the enhanced inflammatory gene expression program associated with IL-10 deficiency in IL-10 knockout mice (170). Ceramide accumulation has also been associated with activation of the inflammasome through stimulation of the ASMase/thioredoxin interacting protein (TXNIP) signaling pathway (173), and it was shown that the short-chain cell permeable octanoyl (C8)-ceramide aggravates endothelial cell damage and increased cell permeability by inducing pyroptosis in human umbilical vascular endothelial cells (174). Moreover, ceramides have been associated with augmented cardiometabolic risk, whereas SMs were protective and associated with reduced cardiometabolic risks (175). In this connection, depletion of SM was found to correlate with inflammation (176) and ceramide accumulation has been associated with the development of cardiovascular disease (177, 178, 179). In fact, the rise in ceramides in the circulation has been linked with development of atherosclerosis (180, 181, 182). Moreover, plasma ceramides and phosphatidylcholines have been described as relevant biomarkers of high blood pressure, with imbalances of these two lipids potentially contributing to the development of hypertension (183). Also of relevance is the finding linking ceramides with formation of abdominal aortic aneurysm. Specifically, it has been demonstrated that degradation of ceramides in platelets by the action of alkaline ceramidase-1 lessens vascular inflammation and abdominal aortic aneurysm development (184). More recently, circulating palmitoyl ceramide has been demonstrated to contribute to inflammation and atherogenesis through activation of Gq-coupled GPCR also involving cysteinyl leukotriene receptor 2 (CYSLTR2) and pyrimidinergic receptor P2Y6 (P2RY6) in endothelial cells and macrophages (185). Another mechanistically relevant action of ceramides is the inhibition of PLD activity, which catalyzes the conversion of phosphatidylcholine, and to a lesser extent other phospholipids, into PA in plasma membranes (186, 187). PA is a bioactive glycerophospholipid capable of regulating vital cellular functions, including actin polymerization and mammalian target of rapamycin (mTOR) activation, which are processes associated with cell migration (188), as well as stimulation of cell proliferation (53, 186, 189, 190, 191, 192), membrane trafficking (193) and cell differentiation (193, 194). Moreover, extracellular vesicles, which can be secreted by normal as well as cancer cells are enriched in PLD2, which produces PA (195, 196). These PA-loaded vesicles can bind to plasma membrane receptors triggering important cellular events, including tumor invasion and metastasis (197). Also, PA can be generated by exogenous PLDs in plasma membranes, a location where PA can participate in signal transduction processes. In this context, we recently demonstrated that exogenous PA, or exogenous (bacterial) PLD, stimulate lung cancer cell migration through a mechanism involving the interaction with lysophosphatidic acid receptor 1 (198). From these observations it can be inferred that the role played by ceramides through inhibition of PLD-derived PA is of paramount importance in cell biology, particularly for controlling pathological processes that are associated with inhibition of cancer cell growth and dissemination. The anticarcinogenic properties of ceramides are well documented in the scientific literature where a number of reports show that many chemotherapeutic drugs produce ceramides as part of their mechanism to induce cell death. Ceramide-producing drugs include anthracyclines, such as doxorubicin (199, 200, 201), cisplatin (202, 203, 204), gemcitabine (205), or the synthetic retinoid fenretinide (N-(4-hydroxyphenyl) retinamide) (206), among others. Moreover, ceramides have been defined as tumor suppressor agents for different types of cancer (207, 208, 209), and are central in research to improve chemotherapy for the treatment of patients with different types of cancer.

Although ceramides can be used as precursors for the synthesis of complex sphingolipids in the ER, ceramide signaling pathways that are mainly triggered in the plasma membrane, can be terminated by the actions of ceramidases, which as mentioned above catalyze the conversion of ceramides into Sph, or by ceramide kinase (CerK), the enzyme that produces C1P through phosphorylation of ceramide (Fig. 1). The latter action is particularly important as C1P is also bioactive, being able to regulate numerous pathophysiological cell functions, as discussed below.

## Ceramide 1-phosphate

C1P is mainly synthesized in the Golgi apparatus where ceramide that is transported from the ER by CERT is phosphorylated by CerK. C1P can also be found in the perinuclear region suggesting that CerK might also be present in the proximity of the nuclear envelop, or that C1P might somehow be transported from the Golgi to the nuclear membrane. In this regard, a specific C1P transfer protein (CPTP) was identified (210). CPTP has been reported to carry C1P from the Golgi apparatus to the plasma membrane and other organelles where it could regulate different cell functions (210, 211, 212). The molecular mechanisms whereby CPTP extracts and inserts C1P into membranes is unclear but processes involving disruption of hydrophobic lipid-membrane contacts and lowering the activation of free energy barrier for passive lipid desorption have been proposed (213).

Until recently, the only mechanism for the biosynthesis of C1P in mammalian cells was upregulation of the ceramide/CerK pathway. CerK was first found in the brain tissue (214) and later identified in human leukemia HL-60 cells (215). However, the observation that CerK KO mice were able to produce significant amounts of C1P led to the discovery of a new C1P biosynthetic pathway, which involved phosphorylation of ceramide by diacylglycerol kinase-ζ (DAGKζ) (216), although given the small ability to synthesize C1P, this enzyme may not be the only missing source in C1P production. An alternative route for C1P biosynthesis might be the SMase D pathway, which produces C1P by precise degradation of SM in some arthropods, including spiders of the genus Loxosceles, such as *Loxosceles arizonica, Loxosceles gaucho, or Loxosceles reclusa* (217, 218), or some bacteria, such as *Vibrio damsella*, *Corynebacterium tuberculosis*, or *Archanobacterium haemolyticum* (219, 220, 221). However, no SMase D has so far been identified in mammalian tissues.

**Figure 4:**
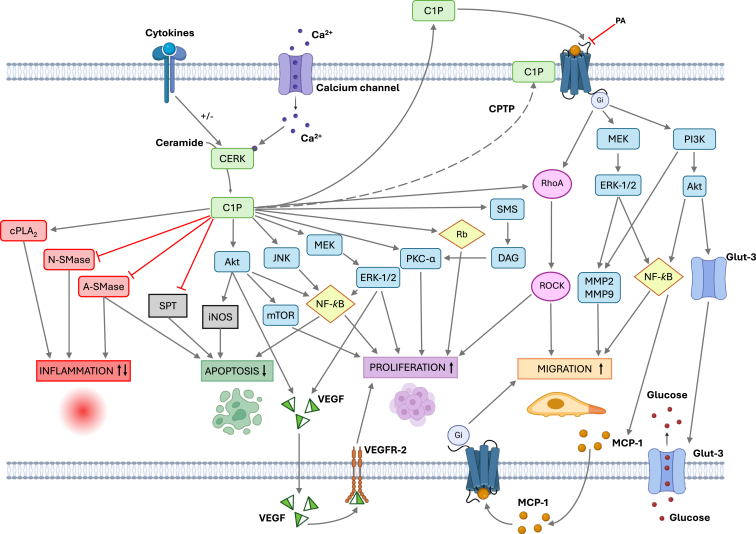
**Biological actions of C1P in mammalian cells.** C1P is synthesized intracellularly by the action of ceramide kinase (CerK) leading to regulation of cell proliferation, apoptosis, and inflammation. C1P is also found in the extracellular milieu, being able to interact with a specific membrane-binding site (possibly a GPCR) that is coupled to Gi proteins. Binding of C1P to its putative receptor leads to regulation of cell migration through upregulation of metalloproteinases 2 and 9 (MMP 2 and 9), also implying the release of macrophage chemoattractant protein 1 (MCP1). Also, extracellular C1P promotes glucose uptake through a mechanism involving stimulation of the GLUT-3 glucose transporter in macrophages. C1P, ceramide 1-phosphate; GPCR, G protein–coupled receptor.

Initial studies using short chain C1P (acetyl, C2-C1P and octanoyl, C8-C1P) demonstrated that phosphorylated ceramides are mitogenic agents, as they were able to significantly stimulate DNA synthesis and cell division in cultured rat-1 fibroblasts (222). Subsequent studies using natural, long chain, C1P in cultured NIH 3T3 rat fibroblasts confirmed the mitogenic activity of C1P (223). The mechanisms whereby C1P stimulated cell division involved phosphorylation (activation) of various cell signaling kinases, including ERK1, ERK2, Akt, c-Jun N-terminal kinase (JNK), PKCα, glycogen synthase kinase-3 (GSK-3) or mTOR (224, 225, 226, 227). Also, non-kinase proteins, including SM synthase (228), retinoblastoma (225), vascular endothelial cell growth factor (229), or production of low levels of reactive oxygen species (230) were involved in the stimulation of cell proliferation by C1P (Fig. 4). In a follow-up study, it was proposed that an alternative way by which C1P increased cell number was through inhibition of apoptotic cell death. Specifically, incubation of primary bone marrow-derived macrophages (BMDMs) in the absence of monocyte/macrophage-colony stimulating factor (M-CSF), a cytokine that is essential for maintaining BMDM viability and growth, caused cell death by apoptosis, and this effect was completely overcome by C1P (231). Apoptotic BMBM showed increased levels of proapoptotic ceramides, which were generated by upregulation of ASMase when the macrophages were incubated in the absence of M-CSF, and C1P completely abrogated the stimulation of ASMase in these cells. The mechanism by which C1P inhibits ASMase is likely to involve physical interaction of C1P with the enzyme as ASMase activity was also completely inhibited by C1P in cell extracts or cell-free systems (231). Likewise, C1P reduced the accumulation of *de novo* synthesized ceramides in cultured, M-CSF-independent, rat alveolar NR8383 macrophages incubated under apoptotic conditions (absence of serum), an action that involved the blockade of SPT activity (232). The physiological relevance of the prosurvival effect of C1P is underscored by the finding that the intracellular levels of C1P were substantially decreased in the primary BMDM after M-CSF withdrawal. Hence, it may be possible that the depletion of C1P levels could release ASMase or SPT from inhibition, thereby triggering ceramide generation and apoptotic cell death. The antiapoptotic actions of C1P also involved upregulation of PI3K/Akt (233, 234), downregulation of nitric oxide synthase (iNOS) (235), and inhibition of caspases 9 and 3 (231). Interestingly, relatively low levels of C1P were also found in human proinflammatory M1 macrophages compared with anti-inflammatory M2 macrophages in the peritoneal cavity. The latter observation was associated with a reduction in the mobility of M1 macrophages, which could not efficiently leave the peritoneal cavity thereby contributing to the development of chronic inflammation and endometriosis (236). These findings are consistent with previous work showing that C1P induces cell migration in human and mouse macrophages (237, 238, 239), as well as in hematopoietic stem/progenitor cells, coronary artery macrovascular endothelial cells and retinal microvascular endothelial cells (240, 241, 242). Noteworthy, macrophage migration across the peritoneum into the lymphatic vessels is a key factor in the resolution of inflammation (243, 244). Also, it was reported that the removal of inflammatory macrophages involved migration across the mesothelium to the draining lymphatics (244), and that dysregulation of macrophage migration results in chronic inflammatory conditions (245, 246, 247). Interestingly PA, which bears structural similarities to C1P, was able to displace C1P from its membrane-binding site (possibly a receptor) blocking macrophage migration (248), thereby pointing to a relevant role of PA in the regulation of macrophage-mediated inflammatory responses. Moreover, PA inhibited C1P-stimulated glucose uptake in macrophages (Fig. 4), which are highly dependent on glucose for accomplishing their biological activities, (150). The latter action also involved displacement of C1P from its putative receptor and blockade of the C1P-stimulated PI3K/Akt pathway. The C1P receptor was partially characterized and found to belong to the GPCR family of plasma membrane receptors that are coupled to Gi/o proteins (239). The possible implication of the C1P receptor in inflammatory responses is a matter of current investigation.

Concerning inflammation, initial studies by Chalfant et al., showed that C1P stimulated group IV cytosolic phospholipase A2 (cPLA2) activity leading to generation of arachidonic acid and proinflammatory eicosanoids in different cell types (249, 250, 251, 252, 253, 254). Also, it has been recently reported that binding of C1P to cPLA2 diverts arachidonic acid formation to the production of proinflammatory prostaglandins at the expense of 5-hydroxyeicosatetraenoic acid and 5-oxo-eicosatetraenoic acid biosynthesis in a context of wound healing and response to sepsis (255), and that inhibition of CerK reduced the secretion of IL-1β and MCP-1 in human monocytes (256). In line with this observation, it has been recently reported that C1P can act as antimicrobial agent for the treatment of human granulocytic anaplasmosis, an infectious disease caused by *Anaplasma phagocytophilum* that can result in inflammation, shock, sepsis, renal failure, and death (257). However, a number of reports suggest that C1P can exert anti-inflammatory actions in different biological settings and cell types. Specifically, C1P was shown to reduce accumulation of proinflammatory ceramides in macrophages and to inhibit macrophage ASMase (231), an enzyme involved in the promotion of apoptosis that is also implicated in the formation of PAF-mediated pulmonary edema through ceramide generation (125). In this connection, it has been recently reported that C1P alleviates high-altitude pulmonary edema by stabilizing aryl hydrocarbon receptor nuclear translocator-like (ARNTL)-mediated mitochondrial dynamics (258). In addition to these observations, inhibition of ASMase protected mice from developing lung edema and sepsis (259) and blocked invasion of *Neisseria meningitidis* into endothelial cells in brain tissue (260) suggesting that C1P might act as an anti-inflammatory agent. A major finding on this line of investigation was that C1P inhibited cigarette smoke-induced airway inflammation in mice *in vivo* and in human airway epithelial cells and neutrophils from patients with COPD (261). In this concern, C1P was able to potently reduce acute and chronic lung inflammation and the development of emphysema, actions that were associated with inhibition of NSMase and blockade of NF-κB activities in mouse lungs. Moreover, in human airway epithelial cells and neutrophils, C1P was able to reduce the release of the proinflammatory cytokines IL1β, IL-6, keratinocyte chemoattractant protein, and macrophage inflammatory protein (261). Furthermore, C1P was demonstrated to attenuate LPS-induced acute lung injury through prevention of NF-κB activation in human neutrophils (262). Intrapulmonary application of C1P before (prophylactic) or 24 h after (therapeutic) LPS administration, blocked neutrophil trafficking into the lungs of mice, and the production of proinflammatory IL-8 in human neutrophils (262). C1P also inhibited the stimulation of TNFα production by LPS (263, 264), and it was shown that human embryonic kidney HEK 293 cells transfected with human TLR4, had lower levels of active NF-κB when challenged with C1P (263, 264). Consequently, treatment with C1P blocked the secretion of proinflammatory IL-6, IL-8, and IL-1β thereby emphasizing the anti-inflammatory potential of C1P. Also, it has been recently shown that synthetic C8-C1P determines proangiogenic and pro-reparative features in human macrophages restraining the proinflammatory M1-like phenotype (265), and that C1P levels were reduced in bronchoalveolar lavage fluid of individuals exposed to second hand smoke (266). Moreover, it has been suggested that CerK is part of a defense mechanism against oxidative damage in the liver (267). The latter observation supports the findings of previous work showing a relationship between CerK and vitamin E, a well-established antioxidant, in a mouse model of inflammatory nonalcoholic fatty liver disease, in which vitamin E prevented hepatic oxidative stress through restoration of CerK (268). Furthermore, it was reported that knocking down CerK led to enhanced inflammation in mice with ulcerative colitis (269). Moreover, oral administration of the sphingosine analog FTY720, which upregulates CerK and is used for the treatment of multiple sclerosis, also ameliorates Alzheimer´s disease (270) and steatosis in a model of nonalcoholic fatty liver disease (271). The latter finding is consistent with the improvement of steatosis in phosphatidylethanolamine methyl transferase deficient mice where CerK is upregulated (268).

## Concluding remarks

The present review highlights the importance of the bioactive sphingolipids Sph, ceramides, S1P and C1P in cell biology and the highly relevant roles they play in the control of inflammatory responses and inflammation-related diseases. As these sphingolipids are interconvertible, and can often induce opposing effects, regulation of the enzymes that are involved in their metabolism as well as keeping an appropriate balance among them is paramount for the maintenance of cell and tissue homeostasis. Consequently, any alteration in the levels of these metabolites may lead to metabolic dysfunction or disease. In particular, whereas Sph and ceramides can signal for cell growth arrest and apoptosis, S1P and C1P promote cell proliferation and inhibit cell death. Concerning inflammation, the situation is not as clear cut since while ceramides are proinflammatory, S1P and C1P can exert both proinflammatory and anti-inflammatory actions depending upon the cellular context, or the cell type in which they are produced. Noteworthy, C1P reduces emphysema and may potentially be used for treating other lung pathologies including asthma, COPD or lung fibrosis. In addition, C1P and Sph can both exert beneficial effects in the organism as they possess antimicrobial properties that can ameliorate or prevent infection.

It should be noted that not all of the molecular species of a given sphingolipid may exert similar actions. For example, ceramides or ceramide 1-phosphates containing long *N*-linked fatty acyl chains may promote different effects to those elicited by sphingolipids bearing short *N*-fatty acyl chains. For example, C2-C1P was much less potent that C8-C1P or long chain C1P to inhibit ASMase activity (231). Also, the different species of sphingoid bases and the degree of unsaturation of the *N*-linked fatty acids might contribute to changes in the bioactivity of the sphingolipid metabolites. It should also be pointed out that sphingolipid-activated pathways may cross-talk with other metabolic or cell signaling pathways that control inflammatory responses, thereby adding complexity to understanding the roles played by these molecules in disease. Moreover, many of the actions of the bioactive sphingolipids are tissue-specific, which makes it difficult to interpret their role in cell biology. Hence, uncovering novel molecular targets and mechanisms of action of the sphingolipid metabolites are critical for understanding the functions of these molecules in cell homeostasis. Furthermore, the use of animal models and sophisticated lipidomic techniques, including high-resolution accurate-mass spectrometry with high mass resolving power, combined with emerging genomic tools will be imperative for understanding the role played by the bioactive sphingolipids in physiology and pathology. Targeting the bioactive sphingolipids, the enzymes that are responsible for their metabolism, or the receptors these sphingolipids bind to may result in the reduction of metabolic alterations that lead to disease, especially those illnesses that are associated with inflammatory responses.

## Conflict of interest

The authors declare that they have no conflicts of interest with the contents of this article.
